# Deciphering lignocellulose deconstruction by the white rot fungus *Irpex lacteus* based on genomic and transcriptomic analyses

**DOI:** 10.1186/s13068-018-1060-9

**Published:** 2018-03-02

**Authors:** Xing Qin, Xiaoyun Su, Huiying Luo, Rui Ma, Bin Yao, Fuying Ma

**Affiliations:** 1 Key Laboratory for Feed Biotechnology of the Ministry of Agriculture, Feed Research Institute, Chinese Academy of Agricultural Sciences, No. 12 South Zhongguancun Street, Beijing, 100081 People’s Republic of China; 2 College of Life Science and Technology, Huazhong University of Science and Technology, Wuhan, 430074 People’s Republic of China

**Keywords:** *Irpex lacteus*, Lignocellulose, Transcriptomics, Manganese peroxidase, Free radicals

## Abstract

**Background:**

*Irpex lacteus* is one of the most potent white rot fungi for biological pretreatment of lignocellulose for second biofuel production. To elucidate the underlying molecular mechanism involved in lignocellulose deconstruction, genomic and transcriptomic analyses were carried out for *I. lacteus* CD2 grown in submerged fermentation using ball-milled corn stover as the carbon source.

**Results:**

*Irpex lacteus* CD2 efficiently decomposed 74.9% lignin, 86.3% cellulose, and 83.5% hemicellulose in corn stover within 9 days. Manganese peroxidases were rapidly induced, followed by accumulation of cellulase and hemicellulase. Genomic analysis revealed that *I. lacteus* CD2 possessed a complete set of lignocellulose-degrading enzyme system composed mainly of class II peroxidases, dye-decolorizing peroxidases, auxiliary enzymes, and 182 glycoside hydrolases. Comparative transcriptomic analysis substantiated the notion of a selection mode of degradation. These analyses also suggested that free radicals, derived either from MnP-organic acid interplay or from Fenton reaction involving Fe^2+^ and H_2_O_2_, could play an important role in lignocellulose degradation.

**Conclusions:**

The selective strategy employed by *I. lacteus* CD2, in combination with low extracellular glycosidases cleaving plant cell wall polysaccharides into fermentable sugars, may account for high pretreatment efficiency of *I. lacteus*. Our study also hints the importance of free radicals for future designing of novel, robust lignocellulose-degrading enzyme cocktails.

**Electronic supplementary material:**

The online version of this article (10.1186/s13068-018-1060-9) contains supplementary material, which is available to authorized users.

## Background

The white rots are a large group of fungi that can efficiently decompose plant cell wall and utilize all its components including lignin, cellulose, and hemicellulose. The white rot fungi depend mainly on oxidoreductases and glycoside hydrolases for lignin and polysaccharides degradation, respectively [1]. In contrast, brown rot fungi selectively degrade carbohydrates, owing to the lack of ligninolytic enzymes such as class II peroxidases and laccases [2–4]. Due to their superior capability in destructing lignocellulose, white rot fungi can be widely used in many biotechnological industries including biofuel and biorefinery, especially in the biological pretreatment of lignocellulosic biomass feedstock [5].

With the advancement in next generation sequencing, whole genome sequencing is widely used to explore the underlying molecular mechanisms of lignocellulose degradation by white rot fungi [6, 7]. To date, dozens of white rot fungal genomes, such as those from *Phanerochaete chrysosporium* and *Ceriporiopsis subvermispora*, have been sequenced and are publicly available [8]. Comparative genomic studies have revealed that class II peroxidases, dye-decolorizing peroxidases (DyPs), and multiple pathways for hydrogen peroxide (H_2_O_2_) production collectively constitute the ligninolytic enzyme system in white rots [9]. With these achievements, multi-omics analyses are emerging as an integrated approach to deepen our understanding of enzymatic degradation of lignocellulose by white rots. In recent years, there have been reports of using multi-omics to analyze *P. chrysosporium* grown on aspen, pine, and spruce [10–12], *C. subvermispora* on aspen [13], *Pycnoporus coccineus* on aspen and pine [14], *Phlebia radiata* on spruce [15], and *Dichomitus squalens* on aspen, spruce, wheat bran, and cotton seed hulls [16]. These studies mainly focused on gene expression patterns in response to different lignocelluloses or times.

White rot fungi are further grouped into simultaneous and selective lignocellulose degraders [17]. The former class, represented by *P. chrysosporium*, simultaneously removes cellulose, hemicellulose, and lignin. The selective degraders, typified by *C. subvermispora*, instead preferentially destroy lignin rather than cellulose and hemicellulose at the early stage [18]. Correspondingly, cellulase is expressed at a constant but high level, while lignin-degrading enzymes gradually accumulate in *P. chrysosporium* confronting lignocellulose [12]. In contrast, *C. subvermispora* secrets mainly peroxidases oxidizing lignin initially, then switches to carbohydrate active enzymes (CAZymes) acting on cellulose and hemicellulose at the advanced stage [13].

The simultaneous and selective paradigms of white rots are defined primarily based on their degradation patterns associated with woody lignocellulose. Interestingly, *Irpex lacteus* CD2, a white rot basidiomycete with potent lignocellulose-degrading ability [19], displays a degradation pattern similar to the selective paradigm in pretreating the corn stover. *I. lacteus* CD2 preferentially degrades lignin at the early stage, followed by sharply elevated cellulose degradation rate at the advanced stage [19]. The same pattern has also been reported recently for degradation of corn stover by another *I. lacteus* strain Fr. 238 617/93 [20]. Recently, the genome of an *I. lacteus* strain F17 was reported [21]. The secretome of *I. lacteus* strain Fr. 238 617/93 grown on wheat straw has also been studied [22]. These investigations undoubtedly deepen our understanding of lignocellulose degradation by *I. lacteus*. However, with these achievements, the molecular mechanism underlying the selective-like mode of lignocellulose degradation by *I. lacteus* and its implication remain unknown. The genomic information of *I. lacteus* has not yet been linked to transcription of genes relevant to lignocellulose degradation. There are no systematic analyses available for *I. lacteus* CD2 (and other strains) grown on corn stover, which is an agricultural residue produced in large amounts and can be used as a feedstock for second-generation biofuels [23]. Herein, the genome of *I. lacteus* CD2 was sequenced and analyzed, combing with biochemical and transcriptomic analyses to elucidate the molecular mechanism on its efficient deconstruction of lignocellulose in corn stover.

## Results

### Genome sequencing of *I. lacteus* CD2 and evolutionary analysis

We sequenced the genome of *I. lacteus* CD2 to a 79-fold coverage using a combination of Illumina HiSeq 2000 platform and the PacBio RS 3rd generation technology. The genome of *I. lacteus* CD2 was distributed in 280 contigs with an assembly of 43.16 Mb. More than half of the total sequence and 69% of the 10,853 predicted genes were in the six largest contigs. Above 92% of the genes could be assigned with a definitive function through homology search against the KEGG, KOG, GO, NR, UniProt, and Tremble databases. The summaries of functional annotations of the genome and the top 50 PFAM domains are shown in Additional files 1 and 2, respectively. Similar to the findings in other white rot fungi, signaling proteins, cytochrome P450 monooxygenases (P450s), and major facilitator superfamily (MFS) transporters constituted the largest families and played important roles in the biosynthesis and transportation of metabolites [24, 25]. Thirty-five sugar transporters, members of the MFS transporters indispensable for cellulose and hemicellulose utilization, were among the top domains in *I. lacteus* CD2 (Additional file 2).

**Fig. 1:**
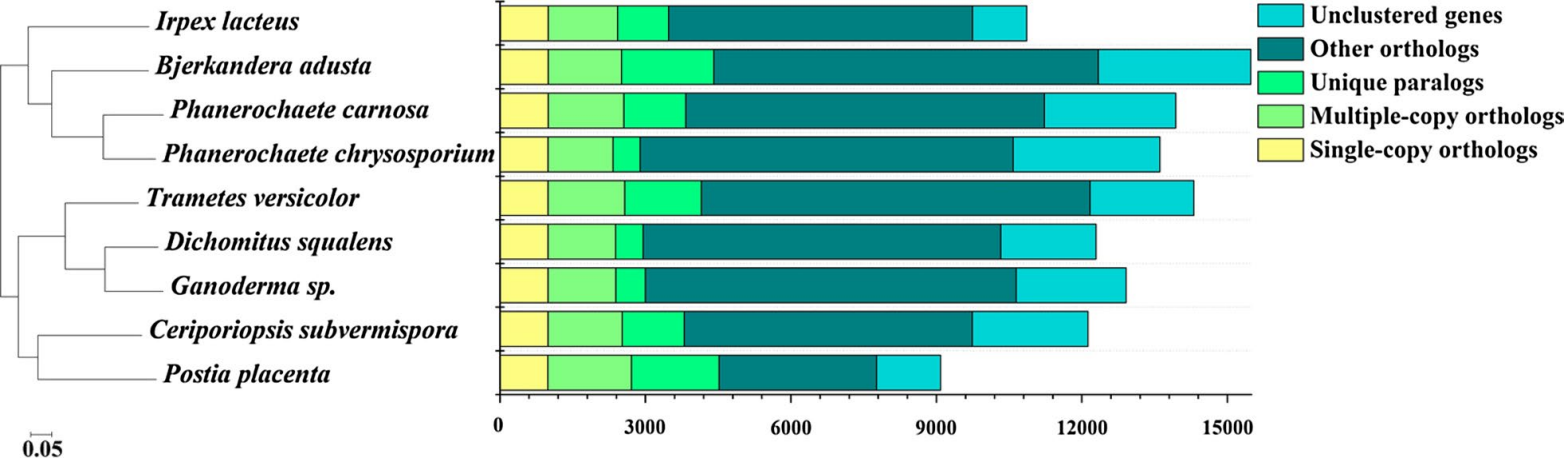
Phylogenomic analysis of *I. lacteus* CD2 with other Polyporales white rots. The representative fungi analyzed in this comparison were *Bjerkandera adusta*, *Trametes versicolor*, *I. lacteus*, *Dichomitus squalens*, *Ganoderma* sp. 10597 SS1, *Ceriporiopsis subvermispora* B, *Postia placenta* MAD 698-R, *Phanerochaete carnosa* HHB-10118-Sp, and *Phanerochaete chrysosporium* RP-78. Orthologs: genes in different species that evolve from a common ancestral gene via speciation. Paralogs: genes produced via gene duplication within a genome

The phylogenomic analysis provides an overview of evolutionary relationship with different strains, serving as a roadmap for its future studies. 987 single-copy orthologous genes were identified in *I. lacteus* CD2 and the other eight sequenced Polyporales. The amino acid sequences of these orthologs were aligned using MAFFT for phylogenetic reconstruction. Maximum likelihood analysis of these genes resulted into a tree with nearly identical topologies (Fig. 1). In the phylogenetic tree, all the nodes had a 100% maximum likelihood bootstrap value. *I. lacteus* CD2 was evolutionarily most close to the lignocellulose degraders *Bjerkandera adusta*, *P. carnosa*, and *P. chrysosporium*.

#### Lignin-degrading oxidoreductive enzymes

The chemical reactions involved in lignin modification by white rot fungi were mainly oxidoreduction [26, 27]. Sixty-two gene models were predicted to encode lignocellulose-degrading oxidoreductases in *I. lacteus* CD2 (Additional file 3), among which nine were fungal class II peroxidases and four were DyPs. In contrast to *T. versicolor*, *D. squalens*, *Ganoderma* sp., and *C. subvermispora*, *I. lacteus* CD2 lacked laccase (AA1_1), which is able to cleave various kinds of linkage in lignin in the presence of appropriate mediators [28]. However, *I. lacteus* CD2 had DyPs (Additional file 3), which deconstruct phenolic and non-phenolic lignin to facilitate enzymatic saccharification of lignocellulose [29]. In addition to peroxidases, *I. lacteus* CD2 encoded 22 glucose methanol choline (GMC) oxidoreductases (AA3) and seven copper radical oxidases (AA5) involved in extracellular H_2_O_2_ generation (Additional file 3). These GMC oxidoreductases were further classified into four groups: one cellobiose dehydrogenase (CDH, AA3_1), sixteen aryl alcohol oxidases (AAO, AA3_2), four alcohol oxidases (AOX, AA3_3), and one pyranose-2-oxidase (POX, AA3_4).

#### Auxiliary enzymes involved in lignin degradation and metabolism

Meanwhile, various low molecular weight compounds including heme, veratryl alcohol (VA), and oxalate were involved in stimulating ligninolytic enzymes production and depolymerizing lignin [26]. Heme was considered as one critical limiting factor for the production of class II peroxidases and P450s [30, 31]. In *I. lacteus* CD2, heme was predicted to be generated from eight consecutive enzymatic reactions and might be regulated by siroheme biogenesis pathway (Additional file 4). VA also played a significant role in the regulation of synthesis of lignocellulose-degrading enzymes [32]. Presumptively, VA was produced through the non-oxidative phenylalanine degradation and the β-oxidation pathways. The related enzymes of *I. lacteus* CD2 were phenylalanine ammonia lyase, AAO, aryl alcohol dehydrogenase, and aryl aldehyde dehydrogenase [33]. Besides, oxalate was normal fungal metabolites facilitating lignin degradation in multiple ways, including chelating unstable Mn^3+^ ions, producing H_2_O_2_, and lowering pH for optimal performance of the ligninolytic enzymes [26, 34]. Four glyoxylate dehydrogenases and two oxaloacetases were discovered to participate in generating oxalate from glyoxylate and oxaloacetate in *I. lacteus* CD2, respectively (Additional file 5). On the other hand, three oxalate decarboxylases and one oxalate oxidase were predicted to be responsible for decomposition of oxalate.

In addition, P450s are indispensable in intracellular metabolism of lignin metabolites and related compounds, and widely distributed in the white rots [35]. The 130 P450s genes of *I. lacteus* CD2 could be grouped into 8 clans containing 17 families with a large portion of the members originating from gene duplication [36]. *I. lacteus* CD2 showed an expansion of the CYP53 clan P450 members involved in lignin metabolism, compared with that in model white rot fungus *P. chrysosporium* [37, 38].

#### Carbohydrate active enzymes involved in Plant cell wall polysaccharides degradation

*Irpex lacteus* CD2 encoded a total of 273 CAZymes, which could be grouped into 182 glycoside hydrolases (GHs), 66 glycosyltransferases (GTs), 5 polysaccharide lyases (PLs), and 20 carbohydrate esterases (CEs), respectively. The cellulose-degrading GHs in *I. lacteus* CD2 consisted of cellobiohydrolases (GH6 and GH7), endoglucanases (GH5, GH9, GH12, GH44, and GH45), and β-glucosidases (GH1 and GH3). The hemicellulose-degrading GHs were composed of xylanases (GH10), xylosidase (GH43), arabinofuranosidase (GH51), and acetylxylan esterase (CE1).

Except for enzymatic hydrolysis, the oxidative cleavage by lytic polysaccharide monooxygenases (LPMOs) was another important route to promote polysaccharide degradation [39, 40]. *I. lacteus* CD2 encoded 17 LPMOs potentially involved in stimulating cellulose and/or hemicellulose degradation [41]. Fifteen of these LPMOs have a C-terminal extension appending to the AA9 core domain with the lengths ranging from 15 to 121 amino acids and four LPMOs have an additional carbohydrate-binding module 1 (CBM1) that might display higher cellulose degradation capability [42].

Interestingly, the CAZyme genes were non-randomly distributed in the *I. lacteus* CD2 genome. Instead, they existed in cluster, particularly for those enzymes belonging to the same CAZyme family. These included a triplet of GH10, a triplet of GH43, a pair of GH5, and a pair of GH18 genes (Additional file 6). None of these adjacent genes appeared to be paralogs, again indicative of frequent gene duplication events during evolution of *I. lacteus* CD2.

#### Genes for generating free radicals involved in lignocellulose degradation

Different types of free radicals, mainly the hydroxyl radical, fatty acid peroxyl radicals, carboxylate-derived radicals, and superoxide radical, are well known for their implication in lignocellulose degradation [43, 44]. In particular, the hydroxyl radical produced in Fenton reaction by Fe^2+^ and H_2_O_2_ is highly reactive, attacking both lignin and cellulose [28]. Iron transportation and reduction hence significantly affect the rate of Fenton reaction. Genes encoding siderophore iron transporters, ferroxidase–permeases, reductase/permeases, and manganese transporters, all of which contribute to iron uptake, were found in the genome of *I. lacteus* CD2 (Table 1). In addition, genes coding for CDH, glycopeptide, benzoquinone reductase, and quinate permease, which are involved in multiple pathways for Fe^3+^ reduction [45–47], were identified (Table 1). Fatty acid peroxyl radicals and carboxylate-derived radicals are generated from chelated Mn^3+^-mediated peroxidation of unsaturated lipid or oxidative decarboxylation of organic acids, respectively [43]. The genes related to fatty acid and oxalate metabolisms are listed in Table 1.

**Table 1:** Genes and their transcription levels relating to metabolism of iron, oxalate, and unsaturated fatty acid, which are involved in free radical generation in *I. lacteus* CD2

Metabolic pattern	Gene	Putative function	FPKM			
			Glu3d	Glu6d	LC3d	LC6d
Iron transportation						
High affinity						
Siderophore-dependent	0809.78	Siderophore iron transporter	77.7	32.8	12.7	47.1
Reductase/ferroxidase-dependent	0809.292	Ferric reductase	113	46.0	29.5	160
	0811.747	Ferric reductase	131	129	138	191
	0809.825	Ferroxidase Fet3	131	5.11	9.88	3.45
	0809.824	Permease Ftr1	158	8.63	4.10	4.12
Low affinity						
Reductase/permease-dependent	0807.394	Manganese transporter SMF2	17.5	36.7	17.7	6.32
	0925.691	Manganese transporter pdt1	54.7	148	25.2	16.4
Iron reduction	0811.251	Cellobiose dehydrogenase	5.54	3.81	42.9	150
	0810.454	Glycopeptide	730	2764	3695	3820
	0809.299	1,4-Benzoquinone reductase	1392	4844	966	396
	0809.746	NADH-quinone oxidoreductase	198	404	537	271
	0808.232	Quinate permease	19.3	27.1	53.0	35.3
	0808.329	Quinate permease	1.66	2.37	91.6	81.5
	0821.194	Quinate permease	137	170	1218	3500
Oxalate biosynthesis	0811.539	Oxaloacetase	7.83	207	103	77.2
	0807.332	Oxaloacetase	50.1	291	277	264
	0811.538	Glyoxylate dehydrogenase	5.21	174	6.35	7.59
	0810.540	Glyoxylate dehydrogenase	36.6	55.0	106	66.7
	0809.407	Glyoxylate dehydrogenase	57.6	66.5	187	127
	0807.465	Glyoxylate dehydrogenase	28.9	78.2	116	69.3
Oxalate degradation	0806.66	Oxalate oxidase	1231	825	33.0	2.66
	0808.407	Oxalate decarboxylase	2.61	7.41	4.53	7.58
	0816.66	Oxalate decarboxylase	131	160	2.26	8.10
	1079.217	Oxalate decarboxylase	3.95	8.49	4.20	4.70
Unsaturated fatty acid biosynthesis	0925.1168	Delta(12) fatty acid desaturase	24.5	14.1	1.15	1.41
	0925.1167	Delta(12) fatty acid desaturase	813	950	90.0	106
	0816.230	Delta(9) fatty acid desaturase	474	798	74.0	139

### Temporal changes in lignocellulose-degrading enzyme activities of *I. lacteus* CD2 grown in lignocellulose medium

**Fig. 2:**
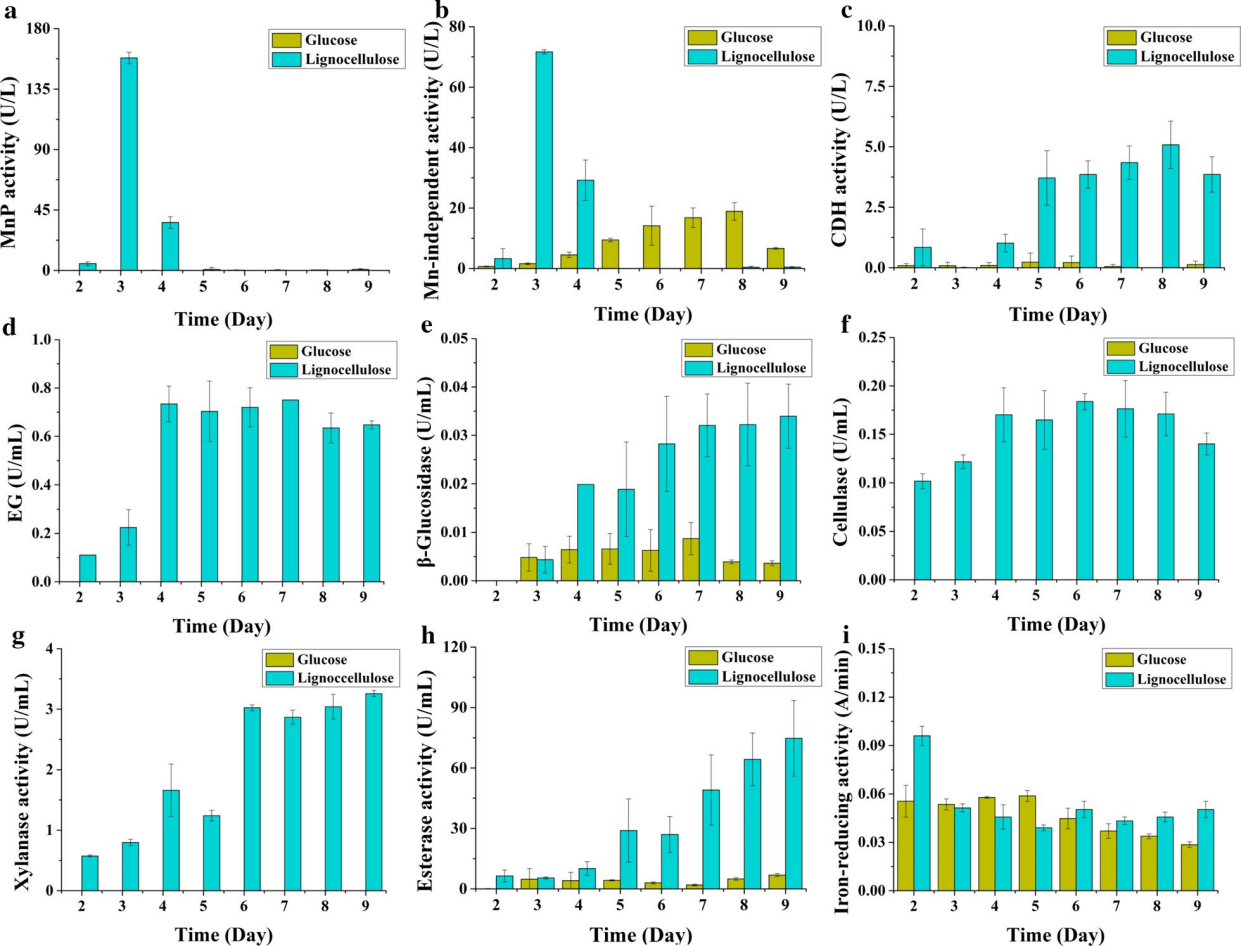
Time-course biochemical analyses of the lignocellulose-degrading enzyme activities of *I. lacteus* CD2 cultured in Kirk’s media containing corn stover or glucose as carbon source. **a** MnP activity; **b** Mn-independent peroxidase activity; **c** CDH activity; **d** endoglucanase activity; **e** β-glucosidase activity; **f** overall cellulase activity; **g** xylanase activity; **h** feruloyl esterase activity; **i** iron-reducing activity. *MnP* manganese peroxidase, *CDH* cellobiose dehydrogenase, *EG* endoglucanase

*Irpex lacteus* CD2 was grown in liquid medium with ball-milled corn stover as carbon source. Since the accessibility of ball-milled lignocellulose was substantially increased [13], *I. lacteus* CD2 rapidly grew and efficiently decomposed 74.9% lignin, 86.3% cellulose, and 83.5% hemicellulose in corn stover within 9 days. Lignocellulose strongly induced expression of manganese peroxidases (MnPs), which are important enzymes involved in lignin degradation by white rots [12, 13, 48], reaching maximal activities of 158 U/L (Fig. 2a) on day 3. Manganese-independent peroxidase (MIP) activity was 72 U/L (Fig. 2b). The activities of MnP and MIP sharply declined afterwards and completely diminished on day 5. No lignin peroxidase (LiP) and DyP activity was detected throughout the cultivation. CDH is an enzyme with multiple roles in both lignin and cellulose degradation [49, 50]. Minor CDH activity was observed from day 2 to day 4, which sharply increased on day 5 and then slowly ascended (Fig. 2c). On the contrary, for plant cell wall polysaccharides degrading enzymes, the endoglucanase (EG) activity gradually increased to a maximal level on day 4 and remained nearly constant in the following days (Fig. 2d). The activity of β-glucosidase (BG) grew from low (day 3 and before) to intermediate (days 4–5) and then to high (days 6–9) (Fig. 2e). The overall cellulase and xylanase performance was similar to that of EG (Fig. 2f, g). Expression of feruloyl esterase, an enzyme cleaving the ferulic acid linking heterogenous xylan and lignin [51], was similar to BG with the change of the intermediate stage to days 5–6 (Fig. 2h). Moreover, we also noticed that the iron-reducing activity in lignocellulose medium was highest at the very early stage (day 2) (Fig. 2i), suggesting that hydroxyl radical was involved in lignin modification by *I. lacteus* CD2 [44].

### Temporal changes in expression of lignocellulose-degrading enzymes based on transcriptomic analysis

**Fig. 3:**
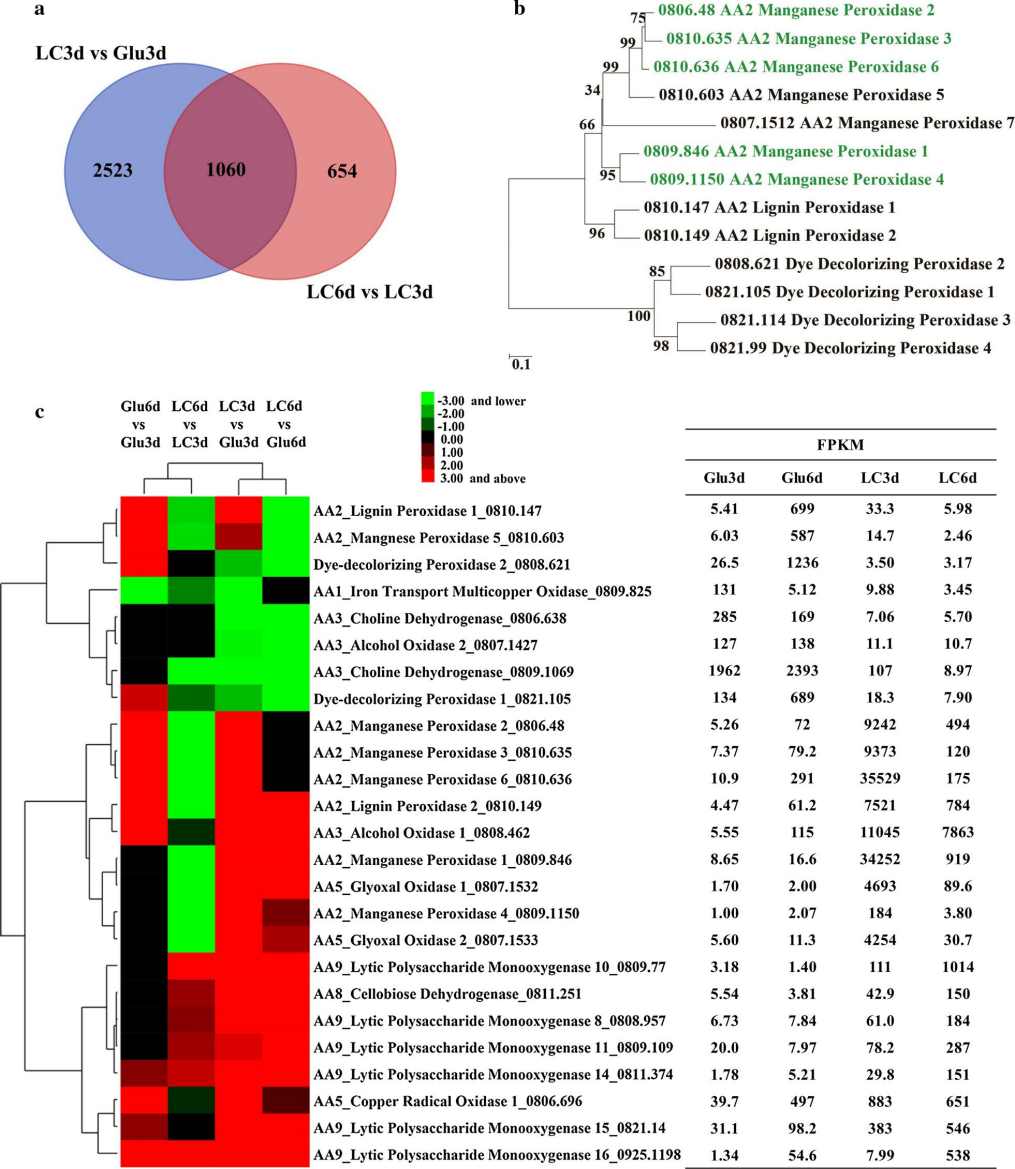
Different expression profiles of ligninolytic enzyme genes. **a** Venn diagram showing differentially expressed genes by comparing LC3d versus Glu3d (Blue) and LC6d versus LC3d (Red). The numbers of gene with transcript levels changing ≥ 2-fold were shown. LC3d: lignocellulose, day 3; LC6d: lignocellulose, day 6; Glu3d: glucose, day 3; Glu6d: glucose, day 6. **b** Phylogenetic analysis of the class II peroxidases and DyPs identified from the genome of *I. lacteus* CD2. The five MnP genes up-regulated in lignocellulose culture (day 3) were highlighted in green. **c** Gene expression profiles for the ligninolytic enzymes associated with lignin modification under the four culturing conditions

Based on the above biochemical results, the total RNAs were extracted from the mycelia of *I. lacteus* CD2 cultured in media containing either lignocellulose or glucose on day 3 and day 6, respectively, and used for RNA-seq transcriptomic analysis. Corresponding to the temporal expression of ligninolytic enzymes and polysaccharides degrading enzymes, the expression levels of a large number of genes were significantly altered (≥ 2-fold) (Fig. 3a and Additional file 7). Comparing the transcriptome of LC3d (lignocellulose, day 3) with Glu3d (glucose, day 3) and LC6d (lignocellulose, day 6) with LC3d by GO analysis, the differently expressed genes were enriched in oxidoreductases and hydrolases (Fig. 3b, c, Table 2 and Additional file 8). KEGG analysis indicated that glyoxylate and phenylalanine metabolism pathway genes involved in degradation of lignin were enriched at the early stage (Additional file 9).

**Table 2:** Genes and their transcription levels relating to plant cell wall polysaccharides degradation under different culture conditions in *I. lacteus* CD2

Substrate preference	Family	Putative function	SignalP	Gene	FPKM			
					Glu3d	Glu6d	LC3d	LC6d
Cellulose	GH3	Beta-glucosidase	Yes	0931.133	53.1	61.6	48.1	107
	GH5	Endoglucanase	Yes	0808.758	5.53	8.30	24.5	33.5
			Yes	0816.65	5.53	5.32	27.0	74.1
	GH6	Cellobiohydrolase	Yes	0824.208	38.6	31.0	3409	12,641
	GH7	Cellobiohydrolase	Yes	0809.465	9.97	2.37	186	102
		Endoglucanase	Yes	0818.184	4.72	4.38	15.7	63.4
	GH12	Endoglucanase	Yes	0811.630	2.44	1.96	2.90	67.5
	GH44	Endoglucanase	Yes	0811.593	5.89	6.94	23.1	132
	GH45	Endoglucanase	Yes	0810.565	2.58	2.53	57.8	82.1
Hemicellulose	GH10	Xylanase	Yes	0808.183	1.24	1.70	48.1	194
			Yes	0808.184	0.48	1.97	2275	547
			Yes	0808.185	37.5	24.4	482	14.7
			Yes	0809.107	0.60	0.42	40.0	643
			Yes	0925.1205	6.58	5.08	12.8	67.7
	GH43	Xylosidase/arabinosidase	No	0925.205	4.23	2.63	116	217
	GH51	α-l-arabinofuranosidase	Yes	0806.422	13.5	9.07	6.14	296
			Yes	0808.962	1.66	1.69	14.3	25.2

#### Enzymatic modification of lignin

The ligninolytic enzymes and auxiliary metabolism systems involved in lignin degradation responded rapidly to lignocellulose. Among the lignin-degrading peroxidases, all MnP genes (except MnP7) were highly induced in LC3d but rapidly decreased in LC6d (Fig. 3c), well corresponding to the biochemical analysis. Besides, RT-qPCR of selected genes important for lignocellulose degradation showed similar expression profiles to those of RNA-seq data (Additional file 10). In comparison, no up-regulation was observed for DyP genes in LC3d (versus Glu3d) or LC6d (versus LC3d). Moreover, the transcript level of one LiP gene (0810.149) was high in LC3d and LC6d, but no enzymatic activity could be detected for LiP. These indicated that enzymatic oxidative degradation of lignin by *I. lacteus* CD2 was mainly through MnPs rather than DyPs and LiPs at the early stage. As for H_2_O_2_ generating oxidases, two glyoxal oxidase (GLOX) genes largely accumulated in LC3d but then quickly diminished in LC6d. AOX1 was expressed to a comparably high level in LC3d and LC6d (Fig. 3c).

Low molecular weight compounds such as heme, aromatic compounds, and oxalate play important roles in efficient lignin degradation. Five key genes in the heme biosynthesis pathway were up-regulated in LC3d and the interfering siroheme biosynthesis pathway was repressed (Additional file 4), indicating that heme production was favored under lignocellulosic conditions. Besides, up-regulation of gene encoding phenylalanine ammonia lyase improved the production of VA, which could significantly enhance the synthesis of MnP produced by *I. lacteus* CD2 [52]. Five transcripts for glyoxylate dehydrogenase and oxaloacetase and three transcripts for oxalate oxidase and oxalate decarboxylase were up-regulated and down-regulated in LC3d, indicating that the synthesis and degradation of oxalate were promoted and inhibited, respectively (Table 1 and Additional file 5). Furthermore, three CYP53 P450s, which were implicated in the intracellular metabolism of low molecular weight lignin and its decomposed fragments [53], were significantly up-regulated in LC3d.

#### Plant cell wall polysaccharides degradation

For cellulose degradation, one GH6 cellobiohydrolase was induced in LC3d compared to Glu3d by 88-fold and further increased by 3.7-fold in LC6d (Table 2). The other cellobiohydrolase was induced in LC3d by 18.7-fold but decreased by 1.2-fold in LC6d. However, its transcript abundance was two orders of magnitude lower than the former one (Table 2). Five among the six endoglucanase genes were up-regulated by 3.3–22.4-fold in LC3d, compared with Glu3d, which further increased by 1.4–5.7-fold in LC6d. The rest endoglucanase (0811.630) had its maximal transcription level in LC6d (27.7-fold higher than in Glu3d). The GH3 β-glucosidase had a comparable transcription level in LC3d to that in Glu3d, but its transcript increased by 2.0-fold in LC6d, compared with LC3d. In addition, AA9 LPMOs displayed very similar expression patterns: they were induced in LC3d and their transcript level continued to ascend in LC6d (Fig. 3c), suggestive of their involvement in cellulose degradation [54].

For hemicellulose degradation, two and three GH10 xylanases were highest expressed in LC3d and LC6d, respectively. A GH43 enzyme predicted to degrade xylooligosaccharides into xylose was induced by 27.4-fold in LC3d and the expression further increased by 1.9-fold in LC6d. Two GH51 arabinofuranosidases predicted to cleave the arabinose side chain were expressed at the highest levels in LC6d (Table 2).

#### Free-radical generation

In addition to enzymatic oxidation of lignin, hydroxyl radical produced from Fenton reaction and carboxylate-derived radicals generated from Mn^3+^/oxalate would also be involved in lignin modification by *I. lacteus* CD2. The genes involved in iron transportation and reduction and H_2_O_2_ production responsible for generating hydroxyl radical displayed a complex regulation pattern. For example, the transcripts of CDH, glycopeptide, and quinate permease responsible for extracellular reduction of Fe^3+^ accumulated in LC3d. In contrast, the transcript levels of siderophore iron transporter and permease Ftr1 were lowest in LC3d (Table 1). Apart from hydroxyl radical, carboxylate-derived radicals were another important kind of free-radical system. Organic acids such as oxalate can stimulate oxidation of non-phenolic lignin by MnPs from *I. lacteus* CD2 [55]. The transcripts for MnPs and oxalate-producing enzymes were most abundant in LC3d (Fig. 3c and Additional file 5).

## Discussion

Previous to our study, there was only one brief report on the genome sequence of an *I. lacteus* strain F17 [21]. In this study, we found that the genome of *I. lacteus* CD2 is 43.16 Mb, similar to but slightly smaller than that of formerly sequenced *I. lacteus* F17 (44.36 Mb). Despite this similarity, however, *I. lacteus* CD2 encodes higher numbers of plant cell wall polysaccharide-degrading enzymes, with 182 GHs compared to 161 GHs in *I. lacteus* F17 [21]. In addition, *I. lacteus* CD2 does not have a laccase, but have 7 MnPs, 2 LiPs, and 4 DyPs, while *I. lacteus* F17 bears one laccase, one LiP, 13 MnPs but no DyP. The study of strain F17 focused on detoxification of synthetic dyes but not in pretreatment of lignocellulose [56, 57]. Therefore, it is not known at this time whether the difference in the numbers and amino acid sequences of encoding enzymes by the two strains would impact their efficiency in lignocellulose degradation. The 18S rRNA phylogenetic analysis suggested that *I. lacteus* F17 is closest to *C. subvermispora*, a selective lignin degrader [21]. Taxonomy based on 18S rRNA gene showed a limitation for certain order level [58–60], while the phylogenomic approach could improve phylogenetic accuracy. Our comparative analysis at a phylogenomic level revealed that *I. lacteus* CD2 is very close to the selective white rot fungus *P. carnosa.*

The sequenced and annotated genome of *I. lacteus* CD2 presented here provides an excellent platform for subsequent biochemical and transcriptomic analyses of this fungus grown on lignocellulose. The carbon source used for submerged shake flask fermentation in this study was ball-milled corn stover, whose natural physical structure has been disrupted to some extent. However, the expression pattern of extracellular lignocellulose-degrading enzymes was still very similar to that of this fungus grown in solid-state fermentation (SSF) on corn stover [19]. Both the current submerged fermentation and previous SSF revealed roughly identical expression patterns, with high lignin-degrading enzymes appearing at the early stage and high expression of cellulase at the late stage.

The large accumulation of MnP transcripts and enzymatic activities at the early stage highlights their importance involved in lignin degradation. Indeed, two recombinant MnPs from *I. lacteus* CD2 exhibited Mn^2+^-dependent oxidation ability for phenolic lignin model compounds [55]. These facts are consistent with the finding that supplementing with Mn^2+^ greatly improved the pretreatment efficiency of corn stover by *I. lacteus* CD2 [61]. MnPs are enzymes that can directly attack phenolic lignin but not non-phenolic lignin compounds [62]. However, phenolic lignin represents only a minor part of lignin (~ 10%), the more recalcitrant and high redox-potential non-phenolic lignin constitutes the major part. The apparent paradox could be explained by our recent discovery that certain carboxylates such as malonate, and more importantly those produced in the life cycle of the fungus such as oxalate, can be used by the above-mentioned MnPs from *I. lacteus* to generate free radicals, which can mediate oxidation of non-phenolic lignin compounds [55]. As a supporting evidence, the genes controlling oxalate production and metabolism experienced regulation favoring accumulation of oxalate on LC3d (Additional file 5). The oxalate concentration was in the range of 0.11 ~ 0.13 mg/ml (1.22 ~ 1.44 mM) from LC3d to LC9d, which can be used by MnPs for oxidation of non-phenolic lignin. Since there was no detectable LiP and DyP activity during cultivation, MnPs, in combination with GLOX and AOX, appeared to be main components of the extracellular lignin-degrading enzyme system for *I. lacteus* CD2 growth on corn stover.

Similar to solid-state biological pretreatment of corn stover by *I. lacteus* CD2 [63], Fe^3+^-reducing activity was detected during all the culture periods. It was noteworthy that the peak of Fe^3+^-reducing activity appeared at the very early stage (day 2) even before the MnPs (day 3). Fe^2+^ can react with H_2_O_2_ via Fenton reaction and produce hydroxyl radical, attacking both lignin and plant cell wall polysaccharides [44]. Hydroxyl radical demethoxylated aromatic groups in lignin and introduces hydroxyl groups in non-phenolic lignin [26]. This process is important, which results into cleavages of lignocellulose barrier, thus facilitating subsequent penetration of MnPs and glycoside hydrolases [64]. Interestingly, the Fe^3+^-reducing activity rapidly decreased to normal state from day 3 to day 9 in lignocellulose medium, which was at a level similar to that in glucose medium (Fig. 2i). However, despite the down-regulated Fe^3+^-reducing activity during this period, degradation of lignocellulose by free radical generated through reaction of Fe^3+^ with H_2_O_2_ cannot be excluded. At the early stage (LC3d), a range of AA3 and AA5 auxiliary enzymes were highly enriched (Table 1), providing H_2_O_2_ for both MnPs and Fe^2+^. At the late stage (LC6d), part of AA3 and AA5 enzymes was still highly expressed (e.g. 0806.696 and 0808.462, Fig. 3c). At this stage, cellulase was highly expressed, hydrolyzing cellulose into cellobiose and cellooligosaccharides. These sugars serve as substrates of CDH, which was also highly induced in LC6d (Fig. 3c), and could generate sufficient amounts of H_2_O_2_ for Fe^2+^. As another important source of hydroxyl radicals [65], the transcript of glycopeptide was highly expressed on LC3d and LC6d, as well.

In accordance with biochemical analysis, the transcriptomic study revealed that the transcripts for MnP isoenzymes were highly expressed at the early stage, but decreased over time in lignocellulose culture. The transcripts for cellulase and hemicellulase gradually increased with the time going on. The special expression pattern of genes encoding lignocellulose-degrading enzymes was similar to that of the selective ligninolytic fungus *C. subvermispora* during growth on aspen wood [13]. However, the transcriptomic and biochemical analyses also revealed significant difference in lignin degradation between *I. lacteus* CD2 and *C. subvermispora*. For lignin decomposition, *I. lacteus* CD2 mainly uses MnPs which are assisted by the H_2_O_2_ producers GLOX and AOX, while *C. subvermispora* utilizes laccases and MnPs with AAO. Moreover, the substrate scope of MnPs from *I. lacteus* CD2 expands to the predominant non-phenolic lignin with the aid of oxalate [55], while peroxyl radical, generated through interplay of fatty acid desaturases and MnPs, is implicated to be responsible for cleavage of non-phenolic lignin in *C. subvermispora* [38]. The expression of important genes involved in lignocellulose degradation is also different between *I. lacteus* CD2 and another model white rot fungus *P. chrysosporium*. In *I. lacteus* CD2, the gene encoding glycopeptide related to Fe^3+^ reduction was highly expressed on LC3d and LC6d. The CDH gene involved in Fe^3+^ reduction was also up-regulated on LC6d compared to LC3d (Table 1), which may affect the rate of Fenton reaction. In contrast, the transcript of CDH remains abundant, while those of glycopeptides are negligible in *P. chrysosporium* during all the culture periods [12].

Taken together, based on biochemical, genomic, and transcriptomic analyses, we could propose a model for lignocellulose degradation by *I. lacteus* CD2 in this submerged fermentation. At the very early stage (day 2 and before), when MnPs are to be expressed, *I. lacteus* CD2 up-regulates the Fe^3+^-reducing power. Fe^2+^ reacts with low level of H_2_O_2_ to produce hydroxyl radical for an initial attack and breakage of interlinking lignocellulose network. At the early stage (days 3–4), MnPs are predominantly expressed and directly oxidize phenolic lignin compound. With assistance of mediators such as oxalate, non-phenolic lignin components are also oxidized by MnPs. Cellulase and xylanase are beginning to accumulate, but still not to the highest level. At the late stage (days 5, 6, and after), MnPs disappear, but part of enzymes responsible for H_2_O_2_ generation are highly expressed, which allow production of H_2_O_2_ and subsequent hydroxyl radical. Cellulase and xylanase are abundantly expressed at this stage, decomposing cellulose and hemicellulose at the maximal speed. In a model described previously [66], the lack of enzymes (such as β-glucosidase and β-xylosidase) cleaving oligosaccharides into simple sugars is key for high saccharification efficiency of grass biomass after *I. lacteus* pretreatment. Indeed, our findings support this hypothesis, because in LC3d, the transcription of β-glucosidase and the enzymatic activity were very low. With this in mind, our findings corroborate the idea that selective expression of lignin-degrading enzymes at the early stage could be an additional key factor for high saccharification efficiency of lignocellulose after *I. lacteus* CD2 biopretreatment. Selective removal of lignin at the early stage exposes cellulose ready for enzymatic hydrolysis, while low expression of extracellular β-glucosidase keeps cellulose in long-chain, non-fermentable form. These two characters enable *I. lacteus* a suitable white rot for pretreating grass feedstock for biofuel production with high efficiency. We also notice that the regulation of Fe^3+^-reducing activity, as well as hydroxyl radical generation, is complex. Since high concentrations of free radicals and H_2_O_2_ are harmful for both enzymes and microbial hosts, this implies that, any attempts to harness the power of free radicals for formulating novel, robust lignocellulose-degrading enzyme cocktails should be delicately designed.

## Conclusions

In this study, we sought to decipher the molecular mechanism underlying efficient lignocellulose degradation by *I. lacteus* CD2. The genome annotation indicated that *I. lacteus* CD2 has a full array of enzymes for lignin, cellulose, and hemicellulose degradation. Both biochemical and transcriptomic analyses indicated that the fungus employed a selective strategy for degradation of lignocellulose in this submerged fermentation, a phenomenon reminiscent to that in solid-state fermentation on corn stover. This strategy, in combination with low extracellular glycosidase activity, forms the basis for its high efficiency in biological pretreatment of corn stover for saccharification.

## Methods

### Strain, media and culture conditions

*Irpex lacteus* CD2 was isolated from Shennongjia Nature Reserve (Hubei, China) and preserved in the Institute of Environment and Resource Microbiology, Huazhong University of Science and Technology, Wuhan, China. *I. lacteus* CD2 was maintained at 4 °C on potato-dextrose agar plates. The inoculum was pre-cultured in potato-dextrose broth for 7 days at 28 °C, transferred into the modified Kirk’s medium as 10% (v/v) inoculum, and shaken at 150 rpm. The Kirk’s medium contained: ball-milled corn stover (or glucose) as carbon source, 10 g/L; ammonium tartrate, 0.2 g/L; KH_2_PO_4_, 2 g/L; MgSO_4_·7H_2_O, 0.71 g/L; CaCl_2_, 0.1 g/L; and 70 mL trace element solution. The trace element solution contains NaCl, 1 g/L; CoCl_2_·6H_2_O, 0.184 g/L; FeSO_4_·7H_2_O, 0.1 g/L; ZnSO_4_·7H_2_O, 0.1 g/L; CuSO_4_, 0.1 g/L; H_3_BO_3_, 0.01 g/L; Na_2_MoO_4_·2H_2_O, 0.01 g/L; KAl(SO_4_)_2_·12H_2_O, 0.01 g/L; and nitrilotriacetic acid, 1.5 g/L [13].

### Genome sequencing and assembly

The genomic DNA of *I. lacteus* CD2 was extracted using an improved cetyltrimethylammonium bromide (CTAB) method [67] from the mycelia, which was sequenced using both the Illumina HiSeq 2000 and PacBio RS platforms. For second-generation sequencing technology on HiSeq 2000 platform, the genomic DNA was randomly fragmented using the Covaris S2 ultrasonic system. For paired-end 100-bp sequencing, one 500-bp library was prepared using the NEBNext ultra DNA library Prep Kit for Illumina according to the manufacturer’s instructions. The Illumina sequencing yielded 5.2 G clean data in 51,969,572 quality-filtered paired-end reads. For third generation sequencing on the PacBio RS II platform, the genomic DNA was sheared using ultrasonics and one 10-kb SMRT-bell library was constructed according to the Pacific Biosciences SMRT Sequencing instruction manual. The PacBio sequencing yielded 4.5 G clean data in 396,897 quality-filtered reads from five SMRT cells. The LSC software was first used for PacBio reads error correction with all the Hiseq 2000 short reads. The corrected PacBio RS data were assembled with hierarchical genome assembly process using default parameters [68].

### Gene identification and annotation

To identify gene models in the *I. lacteus* CD2 genome, the genome assembly was first analyzed for simple sequence repeat using the MISA software and masked by RepeatMasker, RepeatProteinMasker, and TRF using the RepBase library. Using the repeat-masked assembly, gene models were predicted using de novo prediction tools (Augustus, GeneMark-ES, and Snap) and homology prediction tool (GeneWise) with default parameters. The de novo and homology-based gene models were merged to form a comprehensive and non-redundant reference gene models by EVM [69]. The predicted gene models were functionally annotated using BLASTp against the National Center for Biotechnology Information non-redundant database, SwissProt, and TrEMBL and were also mapped to functional terms including KOG, GO, and KEGG pathways. The tRNAscan-SE software was used to detect tRNA regions and its secondary structures [70]. The rRNAs were identified with the hidden Markov models using the RNAmmer software [71].

### Phylogenomic analysis

The genome sequences of *I. lacteus* CD2 and eight other Polyporales fungi including *B. adusta*, *C. subvermispora*, *D. squalens*, *Ganoderma* sp., *P. carnosa*, *P. chrysosporium*, *P. placenta*, and *T. versicolor* were collected, and a phylogenetic tree was constructed by using the MAFFT and FastTree software based on the deduced amino acid sequences of orthologous genes [72, 73].

### CAZymes and auxiliary enzymes

All *I. lacteus* CD2 protein models were subjected to a procedure combing BLAST and HMMer3 searches against sequence libraries and HMM profiles derived from the families of GHs, PLs, CEs, GTs, auxiliary activities (AAs), and CBMs featured in the CAZy database. The Class II peroxidases and DyPs were further confirmed by BLAST search against the PeroxiBase database [74].

### Composition analysis

For compositional analysis, the non-treated corn stover and that treated by *I. lacteus* CD2 (on days 3, 6, and 9) were collected by centrifugation and then dried at 60 °C for 3 days. Lignin, cellulose, and hemicellulose components of corn stover were determined according to the procedure described by the National Renewable Energy Laboratory [75]. For each sample, the compositional analysis was performed in triplicate. The degradation percentage of lignin, cellulose, and hemicellulose was calculated using the following formula:

$$ {\text{Degradation}}\,{\text{rate\,(\%)}}\,{ = }\,\left( { 1\, - \,\frac{{{\text{lignin(cellulose,}}\,{\text{or}}\,{\text{hemicellulose)}}\,{\text{content}}\,{\text{in}}\,{\text{treated}}\,{\text{corn}}\,{\text{stover}}\, \times \,{\text{final}}\,{\text{weight}}\,{\text{of}}\,{\text{corn}}\,{\text{stover}}\,{\text{after}}\,{\text{treatement}}}}{{{\text{lignin}}\, ( {\text{cellulose,}}\,{\text{or}}\,{\text{hemicellulose)}}\,{\text{content}}\,{\text{in}}\,{\text{raw}}\,{\text{corn}}\,{\text{stover}}\, \times \,{\text{initial}}\,{\text{weight}}\,{\text{of}}\,{\text{raw}}\,{\text{corn}}\,{\text{stover}}}}} \right)\, \times \, 1 0 0. $$

### Enzymatic assays

For lignin-degrading enzymes, MnP activity was measured by monitoring the oxidation of 1-mM MnSO_4_ (ε_270_ = 11,590/M/cm) at 270 nm, in a buffer containing 50-mM pH 5.0 malonate, 1-mM MnSO_4_, and 0.1-mM H_2_O_2_. One unit of MnP activity was defined as the amount of enzyme that oxidized 1 μmol of Mn^2+^ per min at 25 °C [52]. The Mn^2+^-independent activity was measured by monitoring the oxidation of 1-mM ABTS (ε_420_ = 36,000/M/cm) at 420 nm, in a buffer containing 50-mM pH 5.0 malonate and 0.1-mM H_2_O_2_. One unit of Mn^2+^-independent activity was defined as the amount of enzyme that oxidized 1 μmol of ABTS per min at 25 °C [55]. Iron-reducing activity was determined by forming Fe^2+^–ferrozine complex in the 50-mM pH 4.8 acetate buffer containing 0.3-mM FeCl_3_ and 4-mM ferrozine. One unit of iron-reducing activity was defined as the rate of absorbance increase at 562 nm/min. The CDH activity was determined by oxidation of 0.3-mM 2,6-dichloroindophenol sodium salt (DCPIP, ε_520_ = 68,000/M/cm) at 520 nm in the presence of 30-mM lactose in the 50-mM pH 4.8 acetate buffer. One unit of CDH activity was defined as the amount of enzyme that oxidized 1 μmol of DCPIP per min at 25 °C [76]. For cellulose- and hemicellulose-degrading enzymes, the overall cellulase activity, EG, BG, and xylanase activities were determined according to the method described by Xu et al. [63]. The esterase activity was determined using 1-mM *p*-nitrophenyl butyrate (ε_348_ = 8321/M/cm) at 348 nm in the 50-mM pH 6.0 sodium phosphate buffer. One unit of esterase activity was defined as the amount of enzyme that released 1 μmol of *p*-nitrophenol per min at 25 °C [77].

### Transcriptome sequencing

*Irpex lacteus* CD2 was grown in the modified Kirk’s medium containing corn stover or glucose as carbon source. The total RNA was extracted from mycelia on days 3 and 6 using the TRIZOL reagent (Invitrogen, Waltham, MA) according to the manufacturer’s instructions. The total RNA was sent to Annoroad Genomics (Beijing, China) for sample preparation and sequencing. All samples were in duplicate. The cDNA library was synthesized and prepared for sequencing using the Illumina mRNA-Seq Sample Prep Kit (San Diego, CA). Briefly, first, the poly(A) containing mRNA was purified from total RNA with poly(T) oligo-attached magnetic beads and fragmented. Next, the cleaved RNA fragments were reversely transcribed into first-strand cDNA using reverse transcriptase and random primers, followed by second-strand cDNA synthesis. Then, the DNA fragments were further subjected to end repair, addition of “A” bases, and adapters’ ligation. Finally, cDNA templates were purified and enriched by PCR amplification. The cDNA libraries were run in independent lanes and paired-end sequences of 125 bp were obtained with at least 4-Gb clean data for each sample using the Illumina Hiseq 2500.

### Differential expression analysis

The raw reads were trimmed and filtered using Trimmomatic software to remove adapters and low-quality bases [78]. Then, clean reads were assembled into transcripts using TopHat and Cufflinks with the *I. lacteus* CD2 genome as a Ref. [79]. All sequences of transcripts were extracted from reference sequence using gffread from cufflinks pipeline. The gene expression levels were conducted using the fragments per kilobase of exon per million fragments mapped (FPKM) method [80], and read counts were analyzed for differential expression using edgeR [81] with a *p* ≤ 0.05 and a false discovery rate (FDR) ≤ 0.01.

Reverse transcription-quantitative PCR (RT-qPCR) of selected ligninolytic genes was used for confirmation of RNA-seq data, which was conducted using TransStart Green RCR SuperMix (TransGen) with gene-specific primers (as shown in Additional file 11). The reaction conditions were set as follows: 95 °C 10 min for initial denaturation; 40 cycles of 94 °C for 10 s, 66 °C for 20 s, and 72 °C for 20 s. The relative fold of change in selected ligninolytic genes was analyzed using the 2^−ΔΔCT^ method with the glycerol 3-phosphate dehydrogenase-normalized Ct values [82]. The samples from LC6d were compared to those from LC3d.

## Additional files

**Additional file 1.** Summary of *I. lacteus* CD2 annotations.

**Additional file 2.** Top 50 PFAM domains in *I. lacteus* CD2 genome.

**Additional file 3.** Oxidoreductive enzymes involved in lignocellulose degradation in *I. lacteus* CD2 and selected Polyporales genomes.

**Additional file 4.** Predicted heme biosynthesis pathway and its regulation in *I. lacteus* CD2.

**Additional file 5.** TCA cycle, GLOX cycle, and predicted oxalate metabolism in *I. lacteus* CD2.

**Additional file 6.** Distribution of the genes encoding glycoside hydrolases and enzymes with auxiliary activities on the 10 largest contigs of *I. lacteus* CD2 genome.

**Additional file 7.** Differential expression analysis in comparisons of LC3d versus Glu3d and LC6d versus LC3d.

**Additional file 8.** GO enrichment analysis of differently expressed genes in comparisons of LC3d versus Glu3d (a), LC6d versus LC3d (b).

**Additional file 9.** KEGG enrichment analysis of differently expressed genes in comparisons of LC3d versus Glu3d (a), LC6d versus LC3d (b).

**Additional file 10.** Verifying the differential expression as revealed by RNA-seq for selected lignocellulose-degrading genes by RT-qPCR.

**Additional file 11.** Primers used for RT-qPCR.

## Abbreviations

DyPs: dye-decolorizing peroxidases
H_2_O_2_: hydrogen peroxide
CAZymes: carbohydrate active enzymes
P450s: cytochrome P450 monooxygenases
MFS: major facilitator superfamily
GMC: glucose methanol choline
CDH: cellobiose dehydrogenase
AAO: aryl alcohol oxidases
AOX: alcohol oxidases
POX: pyranose-2-oxidase
VA: veratryl alcohol
GHs: glycoside hydrolases
GTs: glycosyltransferases
PLs: polysaccharide lyases
CEs: carbohydrate esterases
LPMOs: lytic polysaccharide monooxygenases
CBM1: carbohydrate-binding module 1
MnPs: manganese peroxidases
MIP: manganese-independent peroxidase
LiP: lignin peroxidase
EG: endoglucanase
BG: β-glucosidase
GLOX: glyoxal oxidase
SSF: solid-state fermentation
CTAB: cetyltrimethylammonium bromide
AAs: auxiliary activities
DCPIP: 2,6-dichloroindophenol sodium salt
FPKM: fragments per kilobase of exon per million fragments mapped
FDR: false discovery rate
RT-qPCR: reverse transcription quantitative
