# Supplementary material for: Deciphering lignocellulose deconstruction by the white rot fungus Irpex lacteus based on genomic and transcriptomic analyses
Source: Biotechnol Biofuels. 2018 Mar 2;11:58. doi: 10.1186/s13068-018-1060-9 (PMC5833081; doi:10.1186/s13068-018-1060-9)
Supplement: Supplementary file 1 — Additional file 1. Summary of I. lacteus CD2 annotations. [file 13068_2018_1060_MOESM1_ESM.docx]

**Additional file 1.** Summary of *I. lacteus* CD2 annotations

| **KEGG annotation** | | | **KOG annotation** | | | **GO annotation** | | |
| --- | --- | --- | --- | --- | --- | --- | --- | --- |
| **Pathway** | **Number** | **Percentage （%）** | **Classification** | **Number** | **Percentage（%）** | **Term** | **Number** | **Percentage（%）** |
| Carbohydrate metabolism | 404 | 4 | RNA processing and modification | 125 | 1 | Biological process | 3628 | 33 |
| Energy metabolism | 391 | 4 | Chromatin structure and dynamics | 55 | 1 | Cellular component | 1620 | 15 |
| Lipid metabolism | 178 | 2 | Energy production and conversion | 217 | 2 | Molecular function | 5118 | 47 |
| Nucleotide metabolism | 869 | 8 | Cell cycle control, cell division, chromosome partitioning | 82 | 1 | **Total** | **5724** | **53** |
| Amino acid metabolism | 429 | 4 | Amino acid transport and metabolism | 139 | 1 |  |  |  |
| Metabolism of other amino acids | 109 | 1 | Nucleotide transport and metabolism | 34 | 0 |  |  |  |
| Glycan biosynthesis and metabolism | 40 | 0 | Carbohydrate transport and metabolism | 155 | 1 |  |  |  |
| Metabolism of cofactors and vitamins | 154 | 1 | Coenzyme transport and metabolism | 59 | 1 |  |  |  |
| Metabolism of terpenoids and polyketides | 90 | 1 | Lipid transport and metabolism | 212 | 2 |  |  |  |
| Biosynthesis of other secondary metabolites | 48 | 0 | Translation, ribosomal structure and biogenesis | 142 | 1 |  |  |  |
| Xenobiotics biodegradation and metabolism | 415 | 4 | Transcription | 114 | 1 |  |  |  |
| **Total** | **1549** | **14** | Replication, recombination and repair | 132 | 1 |  |  |  |
|  |  |  | Cell wall/membrane/envelope biogenesis | 34 | 0 |  |  |  |
|  |  |  | Cell motility | 4 | 0 |  |  |  |
|  |  |  | Posttranslational modification, protein turnover, chaperones | 382 | 4 |  |  |  |
|  |  |  | Inorganic ion transport and metabolism | 71 | 1 |  |  |  |
|  |  |  | Secondary metabolites biosynthesis, transport and catabolism | 245 | 2 |  |  |  |
|  |  |  | General function prediction only | 565 | 5 |  |  |  |
|  |  |  | Function unknown | 212 | 2 |  |  |  |
|  |  |  | Signal transduction mechanisms | 254 | 2 |  |  |  |
|  |  |  | Intracellular trafficking, secretion, and vesicular transport | 164 | 2 |  |  |  |
|  |  |  | Defense mechanisms | 38 | 0 |  |  |  |
|  |  |  | Extracellular structures | 2 | 0 |  |  |  |
|  |  |  | Nuclear structure | 12 | 0 |  |  |  |
|  |  |  | Cytoskeleton | 66 | 1 |  |  |  |
|  |  |  | **Total** | **3084** | **28** |  |  |  |
