# Supplementary material for: Deciphering lignocellulose deconstruction by the white rot fungus Irpex lacteus based on genomic and transcriptomic analyses
Source: Biotechnol Biofuels. 2018 Mar 2;11:58. doi: 10.1186/s13068-018-1060-9 (PMC5833081; doi:10.1186/s13068-018-1060-9)
Supplement: Supplementary file 2 — Additional file 2. Top 50 PFAM domains in I. lacteus CD2 genome. [file 13068_2018_1060_MOESM2_ESM.docx]

**Additional file 2.** Top 50 PFAM domains in *I. lacteus* CD2 genome

| **Pfam ID** | **Number** | **Pfam Description** | **Pfam ID** | **Number** | **Pfam Description** | **Pfam ID** | **Number** | **Pfam Description** |
| --- | --- | --- | --- | --- | --- | --- | --- | --- |
| PF00069 | 161 | Protein kinase domain | PF00270 | 33 | DEAD/DEAH box helicase | PF05368 | 19 | NmrA-like family |
| PF12937 | 145 | F-box-like | PF00172 | 31 | Fungal Zn(2)-Cys(6) binuclear cluster domain | PF00724 | 18 | NADH:flavin oxidoreductase / NADH oxidase family |
| PF07690 | 138 | Major facilitator superfamily | PF00271 | 31 | Helicase conserved C-terminal domain | PF00125 | 17 | Core histone H2A/H2B/H3/H4 |
| PF00067 | 130 | Cytochrome P450 | PF04082 | 31 | Fungal specific transcription factor domain | PF00583 | 17 | Acetyltransferase (GNAT) family |
| PF00400 | 101 | WD domain, G-beta repeat | PF08240 | 31 | Alcohol dehydrogenase GroES-like domain | PF01185 | 17 | Fungal hydrophobin |
| PF00106 | 90 | Short chain dehydrogenase | PF00005 | 29 | ABC transporter | PF13460 | 17 | NADH(P)-binding |
| PF00651 | 60 | BTB/POZ domain | PF00004 | 28 | ATPase family associated with various cellular activities (AAA) | PF13639 | 17 | Ring finger domain |
| PF00026 | 50 | Eukaryotic aspartyl protease | PF00071 | 28 | Ras family | PF00155 | 16 | Aminotransferase class I and II |
| PF00076 | 50 | RNA recognition motif. (a.k.a. RRM, RBD, or RNP domain) | PF00226 | 28 | DnaJ domain | PF00734 | 16 | Fungal cellulose binding domain |
| PF00248 | 50 | Aldo/keto reductase family | PF00135 | 26 | Carboxylesterase family | PF04140 | 16 | Isoprenylcysteine carboxyl methyltransferase (ICMT) family |
| PF01753 | 50 | MYND finger | PF00450 | 26 | Serine carboxypeptidase | PF05699 | 16 | hAT family C-terminal dimerisation region |
| PF12697 | 47 | Alpha/beta hydrolase family | PF12770 | 24 | CHAT domain | PF10342 | 16 | Ser-Thr-rich glycosyl-phosphatidyl-inositol-anchored membrane family |
| PF00107 | 42 | Zinc-binding dehydrogenase | PF00501 | 22 | AMP-binding enzyme | PF00149 | 15 | Calcineurin-like phosphoesterase |
| PF00646 | 38 | F-box domain | PF00891 | 20 | O-methyltransferase | PF00856 | 15 | SET domain |
| PF01494 | 37 | FAD binding domain | PF00150 | 19 | Cellulase (glycosyl hydrolase family 5) | PF01636 | 15 | Phosphotransferase enzyme family |
| PF00153 | 36 | Mitochondrial carrier protein | PF00171 | 19 | Aldehyde dehydrogenase family | PF00134 | 14 | Cyclin, N-terminal domain |
| PF00083 | 35 | Sugar (and other) transporter | PF00179 | 19 | Ubiquitin-conjugating enzyme |  |  |  |
