# Supplementary material for: Deciphering lignocellulose deconstruction by the white rot fungus Irpex lacteus based on genomic and transcriptomic analyses
Source: Biotechnol Biofuels. 2018 Mar 2;11:58. doi: 10.1186/s13068-018-1060-9 (PMC5833081; doi:10.1186/s13068-018-1060-9)
Supplement: Supplementary file 3 — Additional file 3. Oxidoreductive enzymes involved in lignocellulose degradation in I. lacteus CD2 and selected Polyporales genomes. [file 13068_2018_1060_MOESM3_ESM.docx]

**Additional file 3.** Oxidoreductive enzymes involved in lignocellulose degradation in *I. lacteus* CD2 and selected Polyporales genomes^a^.

|  | ***Irpex lacteus* CD2** | | ***Bjerkandera adusta*** | ***Phanerochaete carnosa*** | ***Phanerochaete chrysosporium*** | ***Trametes versicolor*** | ***Dichomitus squalens*** | ***Ganoderma sp.*** | ***Ceriporiopsis subvermispora*** | ***Postia placenta*** |
| --- | --- | --- | --- | --- | --- | --- | --- | --- | --- | --- |
| AA1_1 | | 0 | 0 | 0 | 0 | 7 | 11 | 16 | 7 | 2 |
| AA1_2 | | 1 | 1 | 1 | 1 | 2 | 1 | 1 | 1 | 1 |
| AA1_3 | | 0 | 1 | 9 | 4 | 1 | 2 | 1 | 1 | 1 |
| AA2 | | 9 | 20 | 11 | 15 | 26 | 12 | 9 | 17 | 1 |
| AA3_1 | | 1 | 1 | 1 | 1 | 1 | 1 | 1 | 1 | 0 |
| AA3_2 | | 16 | 30 | 32 | 34 | 17 | 30 | 27 | 16 | 24 |
| AA3_3 | | 4 | 8 | 4 | 3 | 4 | 4 | 5 | 4 | 5 |
| AA3_4 | | 1 | 1 | 0 | 1 | 1 | 0 | 0 | 0 | 0 |
| AA4 | | 0 | 0 | 0 | 0 | 0 | 0 | 0 | 0 | 0 |
| AA5_1 | | 7 | 7 | 6 | 7 | 9 | 9 | 9 | 3 | 3 |
| AA5_2 | | 0 | 0 | 0 | 0 | 0 | 0 | 0 | 0 | 0 |
| AA6 | | 2 | 4 | 3 | 4 | 1 | 1 | 2 | 0 | 1 |
| AA7 | | 0 | 0 | 0 | 0 | 0 | 5 | 4 | 0 | 0 |
| AA8 | | 0 | 2 | 2 | 2 | 2 | 2 | 2 | 2 | 0 |
| AA9 | | 17 | 28 | 11 | 16 | 18 | 16 | 16 | 9 | 2 |
| AA10 | | 0 | 0 | 0 | 0 | 0 | 0 | 0 | 0 | 0 |
| DyP | | 4 | 10 | 0 | 0 | 2 | 0 | 3 | 0 | 0 |
| Total | | 62 | 113 | 80 | 88 | 91 | 94 | 96 | 61 | 40 |

**^a^**AA1_1 (Laccase), AA1_2 (Ferroxidase), AA1_3 (Laccase-like multicopper oxidase), AA2 (Class II peroxidase), AA3_1 (Cellobiose dehydrogenase), AA3_2 (Aryl-alcohol oxidase/Glucose oxidase), AA3_3 (Alcohol oxidase), AA3_4 (Pyranose oxidase), AA4 (Vanillyl alcohol oxidase), AA5_1 (Glyoxal oxidase), AA5_2 (Galactose oxidase), AA6 (1,4-Benzoquinone reductase), AA7 (Glucooligosaccharide oxidase), AA8 (Iron reductase domain), AA9 (Lytic polysaccharide monooxygenase) and AA10 (Lytic polysaccharide monooxygenase); DyP (Dye-decolorizing peroxidase).
