# Supplementary material for: Deciphering lignocellulose deconstruction by the white rot fungus Irpex lacteus based on genomic and transcriptomic analyses
Source: Biotechnol Biofuels. 2018 Mar 2;11:58. doi: 10.1186/s13068-018-1060-9 (PMC5833081; doi:10.1186/s13068-018-1060-9)

**Additional file 4.** Predicted heme biosynthesis pathway and its regulation in *I. lacteus* CD2. The genes are shown with their numbers in the genome. The heat map displays the relative transcription level for a gene under different culturing conditions. ALAS 5′-aminolevulinic acid synthase, ALAD 5′-aminolevulinic acid dehydratase, PBGD porphobilinogen deaminase, UROS uroporphyrinogen III synthase, UROD uroporphyrinogen III decarboxylase, CPO coproporphyrinogen III oxidase, PPO protoporphyrinogen oxidase, FC ferrochelatase, Met1 uroporphyrinogen-III C-methyltransferase 1, Met8 uroporphyrinogen-III C-methyltransferase 8.


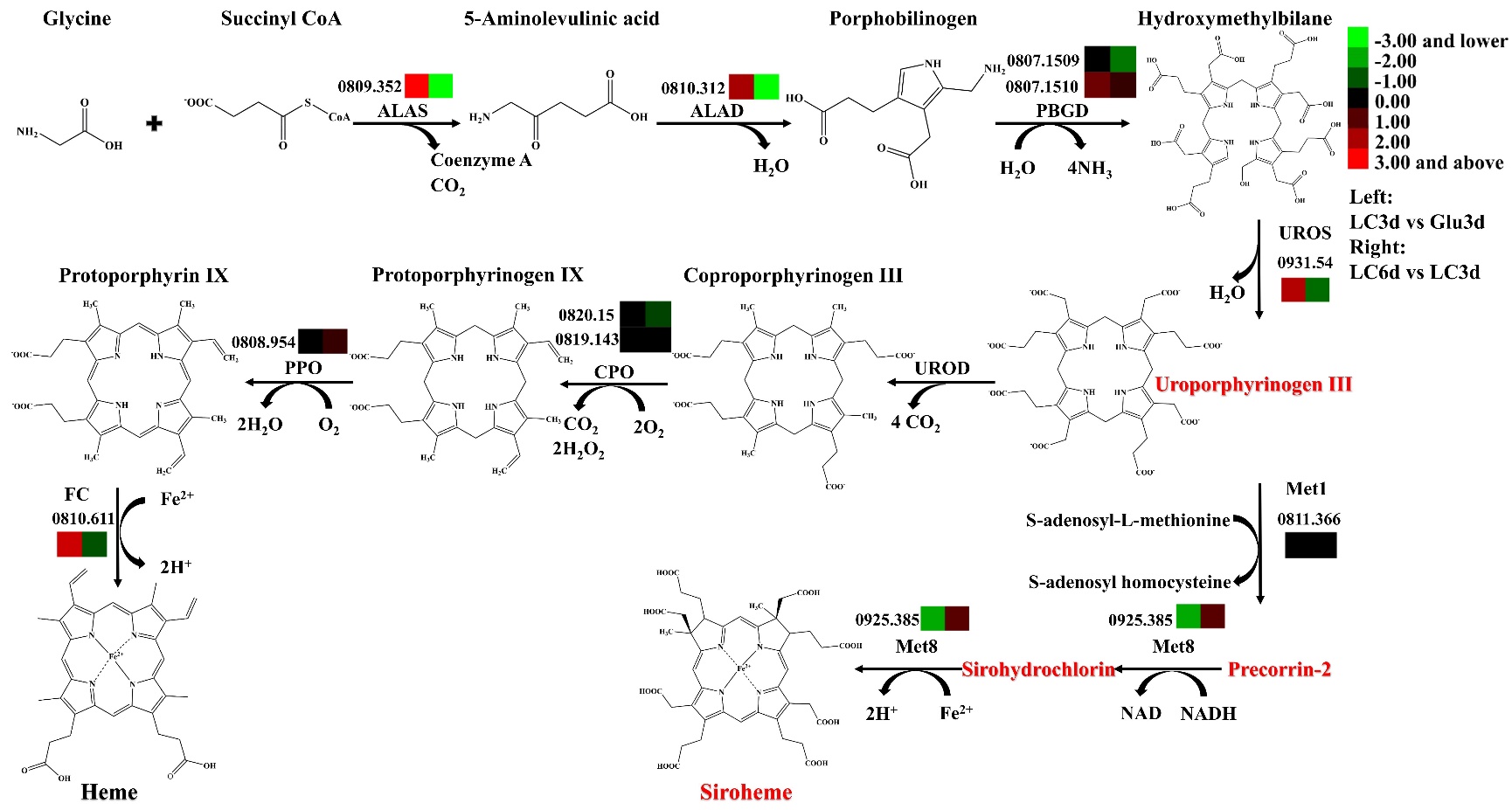

Supplement: Supplementary file 4 — Additional file 4. Predicted heme biosynthesis pathway and its regulation in I. lacteus CD2. [file 13068_2018_1060_MOESM4_ESM.docx]
