# Supplementary material for: Deciphering lignocellulose deconstruction by the white rot fungus Irpex lacteus based on genomic and transcriptomic analyses
Source: Biotechnol Biofuels. 2018 Mar 2;11:58. doi: 10.1186/s13068-018-1060-9 (PMC5833081; doi:10.1186/s13068-018-1060-9)

**Additional file 5.** TCA cycle, GLOX cycle, and predicted oxalate metabolism in *I. lacteus* CD2. The genes are shown with their numbers in the genome. The heat map displays the relative transcription level for a gene under different culturing conditions. PDC pyruvate dehydrogenase; IDH isocitrate dehydrogenase; OGDC 2-oxoglutarate dehydrogenase; SCS succinyl-CoA synthetase; SDH succinate dehydrogenase; MDH malate dehydrogenase; GLDH glyoxylate dehydrogenase.


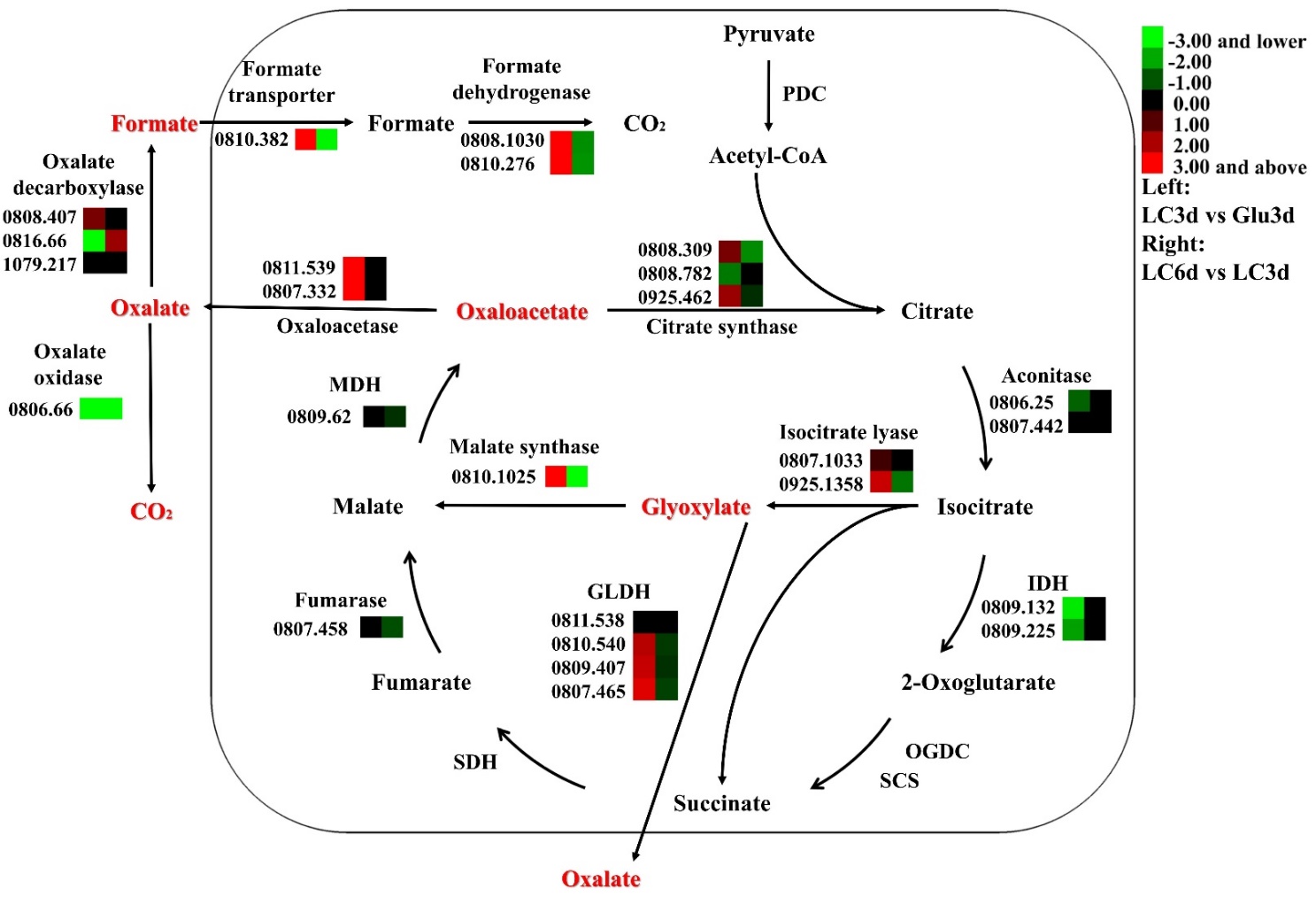

Supplement: Supplementary file 5 — Additional file 5. TCA cycle, GLOX cycle, and predicted oxalate metabolism in I. lacteus CD2. [file 13068_2018_1060_MOESM5_ESM.docx]
