# Supplementary material for: Deciphering lignocellulose deconstruction by the white rot fungus Irpex lacteus based on genomic and transcriptomic analyses
Source: Biotechnol Biofuels. 2018 Mar 2;11:58. doi: 10.1186/s13068-018-1060-9 (PMC5833081; doi:10.1186/s13068-018-1060-9)
Supplement: Supplementary file 6 — Additional file 6. Distribution of the genes encoding glycoside hydrolases and enzymes with auxiliary activities on the 10 largest contigs of I. lacteus CD2 genome. [file 13068_2018_1060_MOESM6_ESM.docx]

**Additional file 6.** Distribution of the genes encoding glycoside hydrolases and enzymes with auxiliary activities on the 10 largest contigs of *I. lacteus* CD2 genome.

**
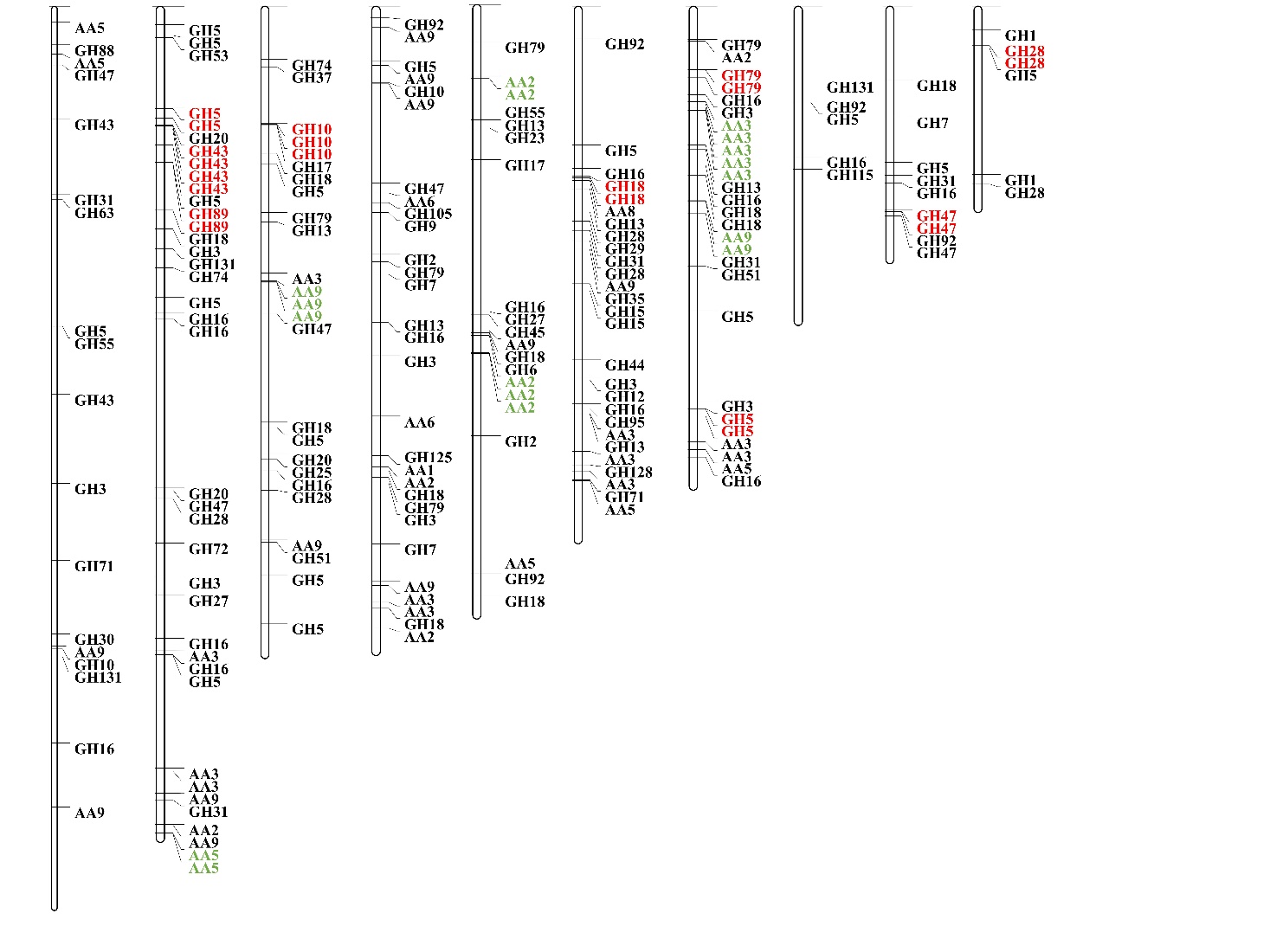
**
