# Supplementary material for: Deciphering lignocellulose deconstruction by the white rot fungus Irpex lacteus based on genomic and transcriptomic analyses
Source: Biotechnol Biofuels. 2018 Mar 2;11:58. doi: 10.1186/s13068-018-1060-9 (PMC5833081; doi:10.1186/s13068-018-1060-9)

**Additional file 8.** GO enrichment analysis of differently expressed genes in comparisons of LC3d versus Glu3d (a), LC6d versus LC3d (b). Biological process: cyan; Cellular component: green; Molecular function: red. *: acting on paired donors, with incorporation or reduction of molecular oxygen; **: acting on paired donors, with incorporation or reduction of molecular oxygen, 2-oxoglutarate as one donor, and incorporation of one atom each of oxygen into both donors.


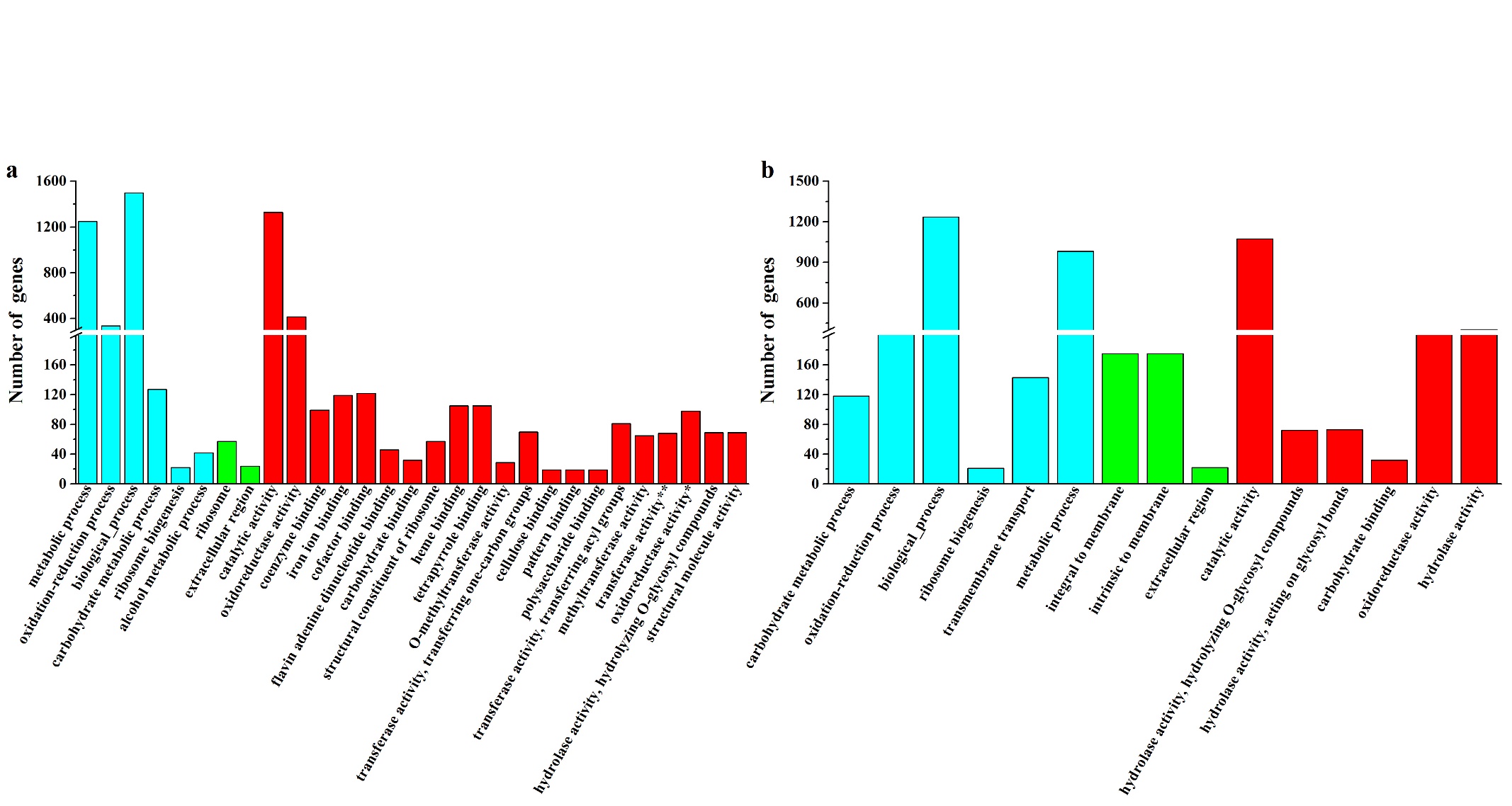

Supplement: Supplementary file 8 — Additional file 8. GO enrichment analysis of differently expressed genes in comparisons of LC3d versus Glu3d (a), LC6d versus LC3d (b). [file 13068_2018_1060_MOESM8_ESM.docx]
