# Supplementary material for: Deciphering lignocellulose deconstruction by the white rot fungus Irpex lacteus based on genomic and transcriptomic analyses
Source: Biotechnol Biofuels. 2018 Mar 2;11:58. doi: 10.1186/s13068-018-1060-9 (PMC5833081; doi:10.1186/s13068-018-1060-9)
Supplement: Supplementary file 10 — Additional file 10. Verifying the differential expression as revealed by RNA-seq for selected lignocellulose-degrading genes by RT-qPCR. [file 13068_2018_1060_MOESM10_ESM.docx]

**Additional file 10.** Verifying the differential expression as revealed by RNA-seq for selected lignocellulose-degrading genes (LC6d versus LC3d) by RT-qPCR.
