# Supplementary material for: Deciphering lignocellulose deconstruction by the white rot fungus Irpex lacteus based on genomic and transcriptomic analyses
Source: Biotechnol Biofuels. 2018 Mar 2;11:58. doi: 10.1186/s13068-018-1060-9 (PMC5833081; doi:10.1186/s13068-018-1060-9)
Supplement: Supplementary file 11 — Additional file 11. Primers used for RT-qPCR. [file 13068_2018_1060_MOESM11_ESM.docx]

**Additional file 11.** Primers used for RT-qPCR.

| **Gene id** | **Primer** | **Nucleotide sequence (5′→3′)** |
| --- | --- | --- |
| 0811.251 | CDH-F | CGGCGGAGGAACATCAATCAAC |
|  | CDH-R | GCCGAGGGATGATCTGTGCT |
| 0809.825 | AA1-F | GCCCAGCGGTACTCTATCCTCGT |
|  | AA1-R | GGGCGGATTTGTTGTAGGTGAT |
| 0809.846 | MnP1-F | GCACCCTCTTCTAGAGTGACATGCAG |
|  | MnP1-R | CACCAAACTTGCCCTCGCGCT |
| 0806.48 | MnP2-F | GCAATCACCAAGCGTGTTGCTTGTC |
|  | MnP2-R | GCCATCGGCACCTCCGCCAC |
| 0810.635 | MnP3-F | GCGATCACAAAGCGGGTTGCG |
|  | MnP3-R | GAGCCGTCAGCACCTCCACCAC |
| 0810.149 | LiP2-F | GCCCCCTCTCCTCTCGTGAGCT |
|  | LiP2-R | GCCACCGAATTTCCCTTGTTGAG |
| 0931.36 | GPD-F | TTCGCTGAGAAGGACCCCGCC |
|  | GPD-R | CAGCAGAGGGAGCAGAGATGACGA |
